# Supplementary material for: TLINet: A defects detection method for insulators of overhead transmission lines using partially transformer block
Source: PLoS One. 2025 Jun 25;20(6):e0327139. doi: 10.1371/journal.pone.0327139 (PMC12194220; doi:10.1371/journal.pone.0327139)
Supplement: Attachment — Submitted filename: Response-to-Reviewers(EN).docx [file pone.0327139.s002.docx]

**Original Manuscript ID:** PONE-D-25-22524

**Original Article Title: “**TLINet: A Defects Detection Method for Insulators of Overhead Transmission Lines Using Partially Transformer Block”

Dear Editor,

Thank you for your feedback. We have carefully revised the manuscript and have removed all text related to funding from the submission, as requested.

Please let us know if any further modifications are needed.

Best regards,

[Yuzhen Zhao]

On behalf of all authors

**Reviewer#1, Concern # 1 (please list here):** Overall, the paper is technically sound but there are some issues in the clarity of the paper. For example, Figures (e.g., Figure 2-5) are referenced but not provided in the text—this limits the reviewer’s understanding of implementation.

**Author response:** Thank you for your recognition of the technical content of this paper and for pointing out the issues regarding its clarity. In response to your comment about Figures 2 to 5 not being properly referenced in the text, we have carefully reviewed and revised the manuscript accordingly. All figures (including Figures 2 to 5) are now explicitly cited in the main text, accompanied by corresponding descriptions and explanations to enhance readers' understanding of the implementation details. We believe these revisions significantly improve the readability and clarity of the paper. We sincerely appreciate your valuable suggestions.

**Author action: For details, please refer to the highlighted section in green on pages 11, 13, 16 and 19.**

**Reviewer#1, Concern # 2 (please list here):** Furthermore, while MBPTB, DyDown, and CGFFN are described, however, the exact architectural parameters (e.g., kernel sizes, number of layers, specific Transformer configs) are sparse or buried in text.

**Author response:** Thank you for your thorough review of our work. Regarding your comment that the architectural parameters of the MBPTB, DyDown, and CGFFN modules (e.g., kernel size, number of layers, specific Transformer configurations) are not clearly described and are partially hidden in the text, we have carefully reviewed and revised the manuscript as follows:

First, we have illustrated the layer configurations of the MBPTB, DyDown, and CGFFN modules in Figure 1.

Second, we have explicitly clarified the number of attention heads used in the Transformer module.

Finally, we have provided the key parameter settings of DyDown and MBCGM, including the kernel sizes.

We believe these additions will help present the implementation details more clearly. Thank you again for your valuable suggestions.

**Author action: For further details, please refer to the Figure 1 and the highlighted section in blue on pages 13, 14, 16, 17 and 19.**

**Reviewer#1, Concern # 3 (please list here):** Although improvements from each module are quantified, statistical significance tests (e.g., standard deviation across multiple runs) are not provided.

**Author response:** Thank you for your valuable comment. In response to your concern regarding the lack of statistical significance tests despite the quantitative improvements of each module, we have made the following revisions in the updated manuscript:

To better evaluate the stability and statistical significance of the model performance, we conducted five independent runs of the ablation experiments. The results are now presented in Table X as mean ± standard deviation (e.g., mAP, Recall).

We believe these additions provide stronger evidence of the effectiveness and robustness of our proposed method. We sincerely appreciate your thoughtful review and constructive feedback.

**Author action: For further details, please refer to the table** X**.**

**Reviewer#1, Concern # 4 (please list here):** There is no discussion of inference time variability or robustness across lighting/weather conditions—relevant to UAV deployment.

**Author response:** Thank you for your valuable comment. We acknowledge that environmental variations such as lighting and weather conditions can significantly affect the inference performance and robustness of models deployed in UAV-based scenarios.

In the revised manuscript, we have added relevant discussions in the **Conclusion** section (highlighted in blue), analyzing the potential impact of various environmental conditions (e.g., lighting intensity, shadows, and weather changes) on model robustness and efficiency. Although our current experiments were conducted under relatively stable lighting conditions, we recognize the critical importance of environmental diversity in real-world applications.

To address this, we have included a qualitative analysis of model predictions under different lighting conditions (such as strong light and shadowed environments) and briefly discussed potential strategies to enhance model robustness.

Thank you again for your insightful suggestion, which has helped us improve the discussion of model performance under real-world deployment scenarios.

**Author action: For further details, please refer to the blue part on page 45.**

**Reviewer#2, Concern # 1 (please list here):** However the references provided are very less, some more references can added. I have also recommended some references you can add them and extend the referencing area.

**Author response:** Thank you for your valuable suggestions regarding the references. We acknowledge that the current number of citations is relatively limited, and have supplemented more relevant literature based on your recommendations to further enrich the reference section. In addition, we have expanded the related content in the manuscript to more comprehensively reflect existing research findings and background. We sincerely appreciate your provided references and constructive comments, which have greatly helped improve the academic depth and completeness of the paper.

We sincerely appreciate your thoughtful recommendation, which has helped us strengthen the completeness and transparency of our manuscript.

**Author action: We have specifically added the following references:**

**Moshayedi A J, Khan A S, Khan Z H, et al. Safeguarding Smart Infrastructure: A Review of Deep Learning Techniques for Automatic Pipeline Defect Detection[J]. Empowering AI Applications in Smart Life and Environment, 2025: 67-92.**

**Moshayedi A J, Khan A S, Yang S, et al. Personal image classifier based handy pipe defect recognizer (HPD): Design and test[C]//2022 7th International Conference on Intelligent Computing and Signal Processing (ICSP). IEEE, 2022: 1721-1728.**

**Xu G, Khan A S, Moshayedi A J, et al. The object detection, perspective and obstacles in robotics: a review[J]. EAI Endorsed Transactions on AI and Robotics, 2022, 1(1).**

**Cheng Y, Liu D. AdIn-DETR: Adapting Detection Transformer for End-to-End Real-Time Power Line Insulator Defect Detection[J]. IEEE Transactions on Instrumentation and Measurement, 2024.**

**Li X, Zhao Y, Zhao Y, et al. MLK-TR: a Multi-branch Large Kernel Transformer for UAV-based Images[J]. Complex & Intelligent Systems, 2025, 11(6): 1-25.**

**Zhou Q, Wang H. CABF-YOLO: a precise and efficient deep learning method for defect detection on strip steel surface[J]. Pattern Analysis and Applications, 2024, 27(2): 36.**

**Tie J, Zhu C, Zheng L, et al. LSKA-YOLOv8: A lightweight steel surface defect detection algorithm based on YOLOv8 improvement[J]. Alexandria Engineering Journal, 2024, 109: 201-212.**

**Reviewer#2, Concern # 2 (please list here):** How does the Multi-Branch Partially Transformer Block (MBPTB) balance the trade-off between capturing global and local features, and what are the benefits of integrating both CNNs and Transformer blocks in the same architecture?

**Author response:** Thank you for your valuable question. The Multi-Branch Partially Transformer Block (MBPTB) achieves an effective balance between global and local feature extraction by splitting the input feature map into two branches, which are processed in parallel by a lightweight CNN module and a Transformer module, respectively. Specifically, the CNN branch focuses on capturing local details and texture information, offering low computational cost and strong local perception capabilities; meanwhile, the Transformer branch models long-range dependencies, enhancing the understanding of global contextual information. Working collaboratively within the same architecture, they preserve CNN’s sensitivity to fine details while leveraging the Transformer’s powerful global semantic modeling ability.

This design not only improves the model’s adaptability to complex backgrounds and multi-scale targets but also effectively controls computational overhead to ensure inference efficiency. By integrating the strengths of both CNN and Transformer, MBPTB can extract multi-scale, multi-level feature information more comprehensively and accurately, significantly enhancing defect detection performance and robustness. We have elaborated the design concept and implementation details of this module in the manuscript. Thank you for your attention.

**Author action: For further details, please refer to the yellow part on pages 12, 13 and 14.**

**Reviewer#2, Concern # 3 (please list here):** In what ways does the Dynamic Downsampling Module (DyDown) differ from traditional downsampling methods, and how does it preserve small-scale defect details while reducing computational complexity?

**Author response:** Thank you for your valuable question. We have provided a detailed design and explanation in the paper regarding the differences between the dynamic downsampling module (DyDown) and traditional downsampling methods. Traditional downsampling methods, such as fixed convolution kernels or single-path pooling operations, can effectively reduce spatial resolution and thus decrease computational load; however, they often lose fine-grained features, especially when detecting small-scale targets in insulator defect images, which negatively impacts detection performance.

To address this, DyDown adopts a multi-path structure and introduces a dynamic convolution mechanism, allowing convolution kernels to adaptively adjust according to the input features, thereby enhancing responsiveness to defects at different scales. Specifically, DyDown first uses average pooling for preliminary compression to preserve local details, then splits the feature map into two paths — one applies dynamic convolution to improve scale adaptability, while the other combines max pooling with dynamic convolution to highlight salient regions and suppress background interference. Finally, the two paths’ features are concatenated to achieve a collaborative expression of local and global information.

This design not only significantly improves the perception of small target defects but also effectively reduces redundant computations while maintaining computational efficiency through reasonable feature branching and dynamic mechanisms. Compared to traditional fixed-structure downsampling modules, DyDown demonstrates superior performance in multi-scale feature representation and detail preservation. Thank you for your attention; we have elaborated on this in detail in the manuscript.

**Author action: For further details, please refer to the yellow part on pages 18 and 19.**

**Reviewer#2, Concern # 4 (please list here):** What specific design principles or mechanisms allow the Context-Guided Feature Fusion Network (CGFFN) to adapt effectively to multi-scale defect detection in complex and cluttered backgrounds?

**Author response:** Thank you for your valuable question. The Context-Guided Feature Fusion Network (CGFFN) effectively adapts to multi-scale defect detection in complex and cluttered backgrounds, relying primarily on the following design principles and mechanisms:

Multi-level Semantic Fusion: CGFFN fuses features from different layers, preserving high-level semantic information while combining it with low-level detailed features, thereby enhancing the model’s ability to recognize multi-scale defects.

Context-Guided Mechanism: The network incorporates a context-guided module that leverages global semantic information to modulate the expression of local features, increasing focus on defect regions and suppressing interference from complex backgrounds, effectively improving the contrast between defects and background.

Dynamic Weight Allocation: CGFFN employs a dynamic weighting mechanism that adaptively adjusts the importance of different feature channels based on the input image’s feature distribution, ensuring that critical defect information is prioritized.

Feature Interaction and Fusion: Through a multi-branch structure, the network enables thorough interaction and fusion of features at different scales, promoting complementary information exchange and enhancing the model’s perception of defects with varying shapes and scales.

These designs enable CGFFN to maintain stable and accurate detection of multi-scale defects under complex and variable background conditions. We have detailed the module’s architecture and working principles in the manuscript. Thank you again for your attention and valuable suggestions.

**Author action: For further details, please refer to the yellow part on pages 15 and 16.**

**Reviewer#2, Concern # 5 (please list here):** Given TLINet’s performance improvements on benchmark datasets (Insulator-DET, CPLID, VOC07+12), what conclusions can be drawn about its generalization capability across diverse data domains and defect types?

**Author response:** Thank you for your important question. Based on the performance improvements of TLINet on multiple benchmark datasets (Insulator-DET, CPLID, VOC07+12), we believe that this method demonstrates good generalization ability across different data domains and defect types. Specifically:

Strong cross-domain adaptability: TLINet has achieved significant performance gains on both power insulator detection datasets (Insulator-DET, CPLID) and the general object detection dataset (VOC07+12), indicating that its design of multi-scale dynamic feature modeling and fusion mechanisms can effectively adapt to varying scenarios and background complexities.

Coverage of diverse defect types: Since these datasets cover various defect shapes and scales, TLINet exhibits high detection accuracy for defects of different sizes and forms, reflecting its strong robustness to diverse defect types.

Stable inference efficiency: Despite involving complex feature fusion structures, TLINet maintains a high inference speed, supporting its potential for rapid response in practical inspection environments.

In summary, TLINet possesses excellent cross-dataset generalization capability and practical application value. We have supplemented the paper with relevant analysis and discussion, and appreciate your valuable suggestions.

**Author action: For further details, please refer to the green part on page 45.**

**Reviewer#2, Concern # 6 (please list here):** What are the practical implications of TLINet’s plug-and-play module design for real-world deployment, and how might future improvements (e.g., handling extreme lighting or occlusion) affect its scalability and adoption in industrial applications?

**Author response:** Thank you for your insightful question. The plug-and-play module design adopted by TLINet holds significant practical value in real-world deployment. Firstly, this design greatly enhances the model’s flexibility and maintainability, allowing different modules to be independently replaced or upgraded according to specific application needs, thereby reducing the complexity of system integration and iteration. Moreover, the plug-and-play architecture facilitates rapid adaptation to diverse detection scenarios and devices, supporting modular deployment and efficient operation.

Regarding future improvements, such as enhancing the capability to handle extreme lighting conditions and occlusions, these will further boost the model’s robustness and stability in complex industrial environments. The improved modules will better adapt to environmental variations, reducing false positives and missed detections, thereby increasing system reliability and user confidence. These advancements will not only expand the model’s applicability but also promote its widespread adoption in industrial automated inspection.

We have supplemented the manuscript with additional explanations on the practical significance of the module design and its potential for future enhancements. Thank you for your valuable suggestions.

**Author action: For further details, please refer to the yellow part on pages 44 and 46.**

**Reviewer#2, Concern # 7 (please list here):** The research objectives are not mentioned in the paper, please mention the objectives.

**Author response:** Thank you for your valuable comments. We have clearly added the research objectives in the introduction section, elaborating on the problems this study aims to address, its innovations, and the expected outcomes, so that readers can better understand the core purpose and contributions of the paper. We appreciate your suggestions, which have helped us further improve the structure and clarity of the manuscript.

**Author action: For further details, please refer to the yellow part on page 3.**

**Reviewer#2, Concern # 8 (please list here):** The research objectives are not mentioned in the paper, please mention the objectives. Aim and Motivation is stated in introduction part. However, it should be explained little more.

**Author response:** Thank you for your valuable comments. We have clearly added the research objectives in the introduction section, elaborating on the problems this study aims to address, its innovations, and the expected outcomes, so that readers can better understand the core purpose and contributions of the paper. We appreciate your suggestions, which have helped us further improve the structure and clarity of the manuscript.

**Author action: For further details, please refer to the yellow part on page 3.**

**Reviewer#2, Concern # 9 (please list here):** Some more related papers can be included in the related work section.

**Author response:** Thank you for your valuable suggestions regarding the references. We acknowledge that the current number of citations is relatively limited, and have supplemented more relevant literature based on your recommendations to further enrich the reference section. In addition, we have expanded the related content in the manuscript to more comprehensively reflect existing research findings and background. We sincerely appreciate your provided references and constructive comments, which have greatly helped improve the academic depth and completeness of the paper.

We sincerely appreciate your thoughtful recommendation, which has helped us strengthen the completeness and transparency of our manuscript.

**Author action: We have specifically added the following references:**

**Moshayedi A J, Khan A S, Khan Z H, et al. Safeguarding Smart Infrastructure: A Review of Deep Learning Techniques for Automatic Pipeline Defect Detection[J]. Empowering AI Applications in Smart Life and Environment, 2025: 67-92.**

**Moshayedi A J, Khan A S, Yang S, et al. Personal image classifier based handy pipe defect recognizer (HPD): Design and test[C]//2022 7th International Conference on Intelligent Computing and Signal Processing (ICSP). IEEE, 2022: 1721-1728.**

**Xu G, Khan A S, Moshayedi A J, et al. The object detection, perspective and obstacles in robotics: a review[J]. EAI Endorsed Transactions on AI and Robotics, 2022, 1(1).**

**Cheng Y, Liu D. AdIn-DETR: Adapting Detection Transformer for End-to-End Real-Time Power Line Insulator Defect Detection[J]. IEEE Transactions on Instrumentation and Measurement, 2024.**

**Li X, Zhao Y, Zhao Y, et al. MLK-TR: a Multi-branch Large Kernel Transformer for UAV-based Images[J]. Complex & Intelligent Systems, 2025, 11(6): 1-25.**

**Zhou Q, Wang H. CABF-YOLO: a precise and efficient deep learning method for defect detection on strip steel surface[J]. Pattern Analysis and Applications, 2024, 27(2): 36.**

**Tie J, Zhu C, Zheng L, et al. LSKA-YOLOv8: A lightweight steel surface defect detection algorithm based on YOLOv8 improvement[J]. Alexandria Engineering Journal, 2024, 109: 201-212.**

**Reviewer#2, Concern # 10 (please list here):** There are some white spaces/gaps in your paper, please fill out those gaps to have a better look for the paper.

**Author response:** Thank you for pointing out the blank sections in the manuscript. We have carefully reviewed the entire paper and filled in all missing parts to ensure the content is complete and coherent. We appreciate your careful review and valuable suggestions.

**Author action: For further details, please refer to the paper.**

**Reviewer#2, Concern # 11 (please list here):** Refer to table 1 inside the text. Explain all the tables briefly inside the text.

**Author response:** Thank you for your valuable suggestion. We have added brief explanations for all tables in the main text, including Table 1, clearly illustrating the key information conveyed and its relevance to our research. These additions help enhance the clarity and logical flow of the paper. We sincerely appreciate your careful review and thoughtful guidance.

**Author action: For further details, please refer to the yellow part on pages 20 and 21.**

**Reviewer#2, Concern # 13 (please list here):** Explain each parameter of the equation/algorithm used.

**Author response:** Thank you for your valuable suggestion. We have supplemented the manuscript with detailed explanations of each parameter used in the equations and algorithms, including their meanings, roles, and practical significance within the network structure. These additions aim to help readers better understand the underlying mechanisms and mathematical formulations. Relevant clarifications have been clearly indicated in the corresponding sections. We appreciate your careful review and thoughtful guidance.

**Author action: For further details, please refer to the blue part on pages 13 and 14.**

**Reviewer#2, Concern # 14 (please list here):** References are not in proper order.

**Author response:** Thank you for pointing out the issue regarding the citation order. We have thoroughly reviewed all the references in the manuscript and have reordered the citations according to the sequence in which they first appear in the text, ensuring their accuracy and compliance with citation standards. We sincerely appreciate your careful review and valuable suggestions.

**Author action: For further details, please refer to the paper.**

**Reviewer#2, Concern # 15 (please list here):** The figures should be explain in more details inside the text.

**Author response:** Thank you for your valuable suggestion. We have revised the manuscript to provide more detailed explanations of all figures within the text, clarifying the meaning of each component, the characteristics of the data presented, and their relevance to the research findings. These improvements enhance the readability and logical coherence of the paper. We sincerely appreciate your careful review and constructive feedback.

**Author action: For further details, please refer to the blue part on pages 16, 17 and 19.**

**Reviewer#2, Concern # 16 (please list here):** Some very little mistakes in grammar, that can be revised.

**Author response:** Thank you for pointing out the minor grammatical issues in the manuscript. We have carefully reviewed and polished the entire text, correcting all identified grammatical errors and improving the overall language quality and readability of the paper. We sincerely appreciate your thorough review and valuable feedback.

**Author action: For further details, please refer to the paper.**

**Reviewer#2, Concern # 17 (please list here):** The conclusion and future scope are stated good. But state what innovative method can be used in the future. State how your approach can help in the relevant field?

**Author response:** Thank you for your positive feedback on our conclusion and for your valuable suggestions. In response, we have expanded the “Conclusion and Future Work” section to outline potential innovative directions for future research, such as integrating cross-modal feature enhancement, lightweight Transformer architectures, and self-supervised learning strategies to improve robustness and generalization under challenging conditions. We have also elaborated on how our proposed approach can contribute to relevant fields, including power line inspection and industrial visual defect detection. Your comments have been instrumental in helping us strengthen the clarity and depth of our paper.

**Author action: For further details, please refer to the yellow and blue part on CONCLUSION Section.**

**Reviewer#2, Concern # 18 (please list here):** The contributions to the field are stated nicely. Some more details can be added in the conclusion part.

**Author response:** Thank you for your positive feedback on our conclusion and for your valuable suggestions. In response, we have expanded the “Conclusion and Future Work” section to outline potential innovative directions for future research, such as integrating cross-modal feature enhancement, lightweight Transformer architectures, and self-supervised learning strategies to improve robustness and generalization under challenging conditions. We have also elaborated on how our proposed approach can contribute to relevant fields, including power line inspection and industrial visual defect detection. Your comments have been instrumental in helping us strengthen the clarity and depth of our paper.

**Author action: For further details, please refer to the yellow and blue part on CONCLUSION Section.**
